# Triglyceride-glucose index as a mediator of body mass index and cardiovascular disease in middle-aged and older Chinese adults: a nationally representative longitudinal cohort study

**DOI:** 10.3389/fendo.2024.1431087

**Published:** 2024-12-23

**Authors:** Ying-Yuan Gan, Lu Zhai, Qian Liao, Rong-Rui Huo

**Affiliations:** ^1^ Department of Scientific Research, Minzu Hospital of Guangxi Zhuang Autonomous Region, Nanning, China; ^2^ Department of Smart Health Elderly Care Services and Management, School of Nursing, Guangxi Health Science College, Nanning, China; ^3^ Department of Epidemiology and Health Statistics, School of Public Health, Guangxi Medical University, Nanning, China; ^4^ Department of Experimental Research, Guangxi Medical University Cancer Hospital, Nanning, China

**Keywords:** cardiovascular disease, triglyceride-glucose index, body mass index, mediator, 4-way decomposition approach, CHARLS

## Abstract

**Background:**

Body mass index (BMI) consistently correlates with the triglyceride-glucose (TyG) index, a marker of insulin resistance, which in turn is linked to heightened cardiovascular disease (CVD) risk. Thus, insulin resistance could potentially mediate the association between BMI and CVD risk. However, few studies have explored this mechanism in the general population.

**Methods:**

We used data from the China Health and Retirement Longitudinal Study, which is an ongoing prospective cohort study. It initially enrolled 7233 middle-aged and older Chinese adults who were free of heart disease and stroke at baseline. The exposure variable was BMI. Incident CVD, defined as self-reported physician-diagnosed heart disease and stroke combined, served as the main outcome.

**Results:**

Of the 7 233 participants (mean [SD] age, 58.93 [9.33] years), 3 415 (47.2%) were men. During the 7 years of follow-up, 1 411 incident CVD cases were identified. Both BMI and TyG index were associated with CVD risk (HR per 1-SD increase: BMI, 1.23; 95% CI, 1.17–1.29; TyG, 1.13; 95% CI, 1.07–1.19). The 4-way decomposition analysis show that, overweight increased CVD risk by 28% (HR [total association], 1.28; 95% CI, 1.14–1.45), with 18.1% (95% CI, 2.2%–34.0%) mediated by TyG index (HR [pure indirect association], 1.05; 95% CI, 1.02–1.09); while obesity increased CVD risk by 91% (HR [total association], 1.91; 95% CI, 1.63–2.23), with 9.5% (95% CI, 2.2%–16.7%) mediated by TyG index (HR [pure indirect association], 1.09; 95% CI, 1.03–1.15). No evidence suggested TyG index modified BMI’s association with incident CVD.

**Conclusions:**

The study revealed that the TyG index was associated to CVD risk and acted as a small partial mediator in the relationship between BMI and CVD among middle-aged and older Chinese adults. Consequently, solely addressing insulin resistance might not significantly mitigate the impact of body weight on CVD. Thus, exploring alternative pathways and potential mediators of CVD risk becomes imperative.

## Introduction

The ongoing challenge of cardiovascular disease (CVD) persists in its impact on global health, affecting both morbidity and mortality rates, and imposing a significant burden on healthcare systems and individual well-being (1, 2). In the last thirty years, the global burden of CVD has surged, with a 92.3% increase in total prevalent cases from 271 million to 523 million and a 53.7% rise in deaths from 12.1 million to 18.6 million between 1990 and 2019 (3). This upward trend is driven by factors such as an aging population and lifestyle changes, including a higher prevalence of obesity, hypertension, and diabetes (1, 4). Although recent studies suggest a potential slowdown in the rise of overweight and obesity in high-income countries (5, 6), there is mounting evidence indicating an acceleration of this epidemic in low- and middle-income countries (7, 8). Notably, obesity globally is associated to an elevated risk of CVD across the general population (9, 10).

Although hemodynamic and metabolic factors have been suggested as factors that influence the relationship between BMI and CVD, the exact mechanisms are not yet fully understood (9, 11). Reduced insulin sensitivity constitutes a potential constituent, as evidence suggests that oxidative stress and inflammation instigated by obesity are intricately associated with the emergence of both localized and systemic insulin resistance (12). Conversely, insulin resistance is implicated in endothelial dysfunction, fostering the development of atherosclerotic plaques through alteration of gene expression patterns related to the estrogen receptor, Hence, it could potentially play a substantial role in the pathogenesis of CVD (13). It is therefore possible that insulin resistance could exert an indirect influence on CVD through BMI. TyG (triglyceride-glucose) index has been verified as a straightforward indicator of insulin resistance based on the logarithmization of glucose levels and fasting triglyceride (14). There has been evidence of a correlation between this test and the euglycemic-hyperinsulinemic clamp test, as well as a similar validity to that of the insulin resistance index calculated from the homeostatic model assessment (15). Given its accessibility and reliable performance, it is convenient for epidemiological studies to use the TyG index to measure insulin resistance as a simple proxy.

Prior studies have combined TyG index and BMI as a TyG-BMI index to examine the association with CVD and outcomes (16–19). Yet, to our knowledge, only one study has formally investigated the TyG index’s role as a mediator in the connection between BMI and incident CVD within a community-based setting, with the majority of participants being coal miners (20). Limitations include population heterogeneity, absence of generalizable, or absence of interaction between BMI and TyG index.

Hence, the objective of this study was to examine whether TyG acts as a mediator or modifier in the relationship between BMI and incident CVD within the general population. Using a causal mediation approach, we disassembled the overall association of BMI with incident CVD into four components: (1) the association unaffected by mediation or interaction, (2) the association influenced solely by interaction, (3) the association driven solely by mediation, and (4) the association influenced by both mediation and interaction.

## Methods

### Study population

This cohort study represents a secondary analysis of the CHARLS dataset, which is an ongoing, nationally representative cohort study. Detailed information regarding the study design is available elsewhere (21, 22). In summary, the study recruited 17708 participants from June 2011 to March 2012. For the purpose of gathering information, participants underwent assessments using standardized questionnaires using a multistage stratified probability proportional-to-size sampling method. The baseline survey achieved an 80.5% response rate. Following the baseline assessment, participants underwent follow-up evaluations every 2 years.

All participants provided written informed consent to participate in the CHARLS study, which was approved by the institutional review board of Peking University. All study protocols were conducted in accordance with the principles outlined in the Declaration of Helsinki (23), and adherence to the Strengthening the Reporting of Observational Studies in Epidemiology (STROBE) reporting guideline was ensured for this study (24).

### Assessment of exposure and mediator

The exposure variable in this study was BMI, calculated from height and weight measurements as weight in kilograms divided by height in meters squared. BMI was categorized according to the Chinese BMI classification (25) as follows: underweight (BMI <18.5 kg/m^2^), normal weight (BMI 18.5–23.9 kg/m^2^), overweight (BMI 24.0–27.9 kg/m^2^), and obesity (BMI ≥28 kg/m^2^). Trained nurses conducted the measurements of height and weight.

The mediator was TyG index, which was calculated as ln [fasting blood glucose (milligrams per deciliter)  ×  triglycerides (milligrams per deciliter)/2) (14) and splitting into quartiles. A colorimetric enzyme assay was used at Capital Medical University’s Youanmen Clinical Laboratory to determine triglycerides and fasting blood glucose levels.

### Ascertainment of outcome

The primary outcome was incident CVD, the secondary outcome were incident stroke events and incident heart disease events. Consistent with prior studies (21, 26, 27), the following standardized questions were used to assess CVD events: “Have you received a diagnosis from a doctor indicating that you have experienced a heart attack, coronary heart disease, angina, congestive heart failure, or any other heart-related conditions?” or “Have you been diagnosed by a doctor with having had a stroke?” Participants who reported either a stroke or heart disease during follow-up were categorized as having experienced a CVD event. The date of CVD diagnosis was recorded between the last interview and the one in which the CVD event was reported (21, 26, 27).

### Covariates

At baseline, trained interviewers used a structured questionnaire to collect information included age, sex, living residence, marital status (categorized as married or other), and educational level (grouped into no formal education, primary school, middle or high school, and college or above), self-reported smoking and drinking status (classified as never, former, or current), self-reported physician-diagnosed medical conditions (including diabetes, hypertension, dyslipidemia, and kidney disease), and the use of medications for these conditions. Metabolic factors comprised fasting plasma glucose, total cholesterol, triglycerides, high-density lipoprotein cholesterol (HDL-C), low-density lipoprotein cholesterol (LDL-C), high-sensitivity C-reactive protein (hsCRP), and serum creatinine. The estimated glomerular filtration rate (eGFR) was calculated using the Chronic Kidney Disease Epidemiology Collaboration’s 2009 creatinine equation (28).

Chronic kidney disease was defined as eGFR <60 mL/min/1.73 m^2^ or self-reported history of chronic kidney disease. Diabetes was defined as fasting plasma glucose ≥126 mg/dL, current use of antidiabetic medication, or self-reported history of diabetes. Dyslipidemia was defined as total cholesterol ≥240 mg/dL, triglycerides ≥150 mg/dL, LDL-C ≥160 mg/dL, HDL-C <40 mg/dL, current use of lipid-lowering medication, or self-reported history of dyslipidemia. Hypertension was defined as systolic blood pressure ≥140 mmHg, diastolic blood pressure ≥90 mmHg, current use of the antihypertensive medication, or self-reported history of hypertension.

### Statistical analysis

Descriptive statistics included mean ± standard deviation (SD) and median with interquartile range (IQR). Categorical variables were depicted as n(%). Baseline characteristics were stratified by TyG index quartiles and compared using appropriate tests: χ² test, analysis of variance, or Kruskal-Wallis rank sum test. Missing data were imputed using the multiple imputation of chained equations method.

**Figure 1:**
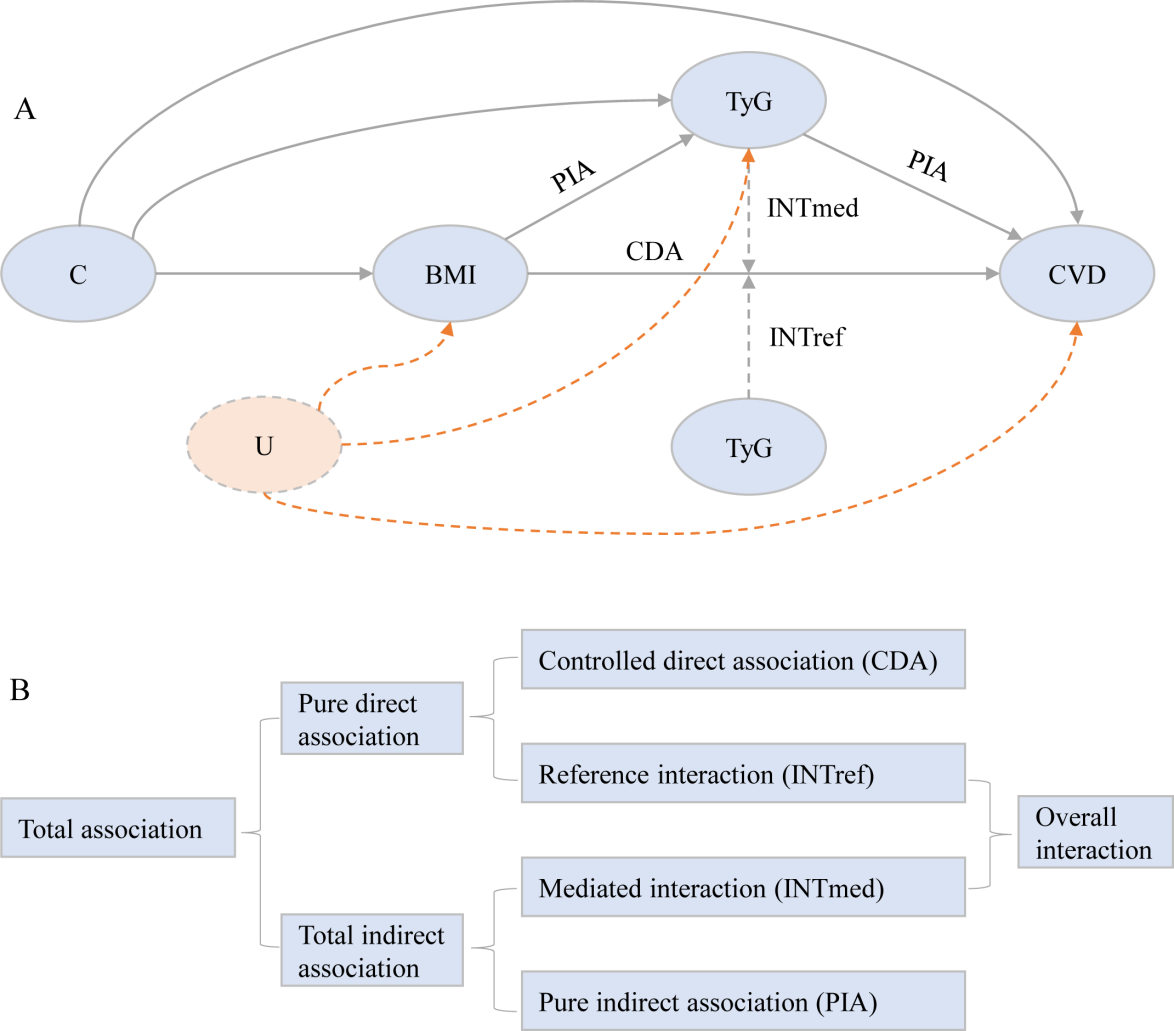
Conceptual model for the analysis of TyG index mediating the association of body mass index with incident cardiovascular disease. **(A)** The figure shows how TyG index could serve as a mediator of the association of body mass index with cardiovascular disease. The direct association between BMI and CVD is also caused by other potential mechanisms, such as hypertension, hypercholesterolemia, and/or diabetes. All statistical models were based on this structure and were adjusted for age, gender, marital status, residence, education level, smoking status, and drinking status. Following the theory of causal graphs, variables such as blood pressure (hypertension, systolic blood pressure, and diastolic blood pressure), cholesterol (total cholesterol, HDL-C, LDL-C, and dyslipidemia), renal function (kidney disease and eGFR), diabetes, glycated hemoglobin, and hsCRP represent alternative pathways that could potentially mediate aspects of the association between BMI and CVD. As such, these variables were not included as covariates in our models. C denotes the potential exposure-mediator, exposure-outcome, and mediator-outcome confounders. U denotes unmeasured confounding, which remains unavoidable in observational research settings. **(B)** Illustration of the 4-way decomposition of total association. The CDA is due to neither mediation nor interaction. The INTref is only due to interaction. The mediated INTmed is due to both mediation and interaction. PIA is only due to mediation. CDA, controlled direct association; INTref, reference interaction; INTmed, mediated interaction; PIA, pure indirect association; BMI, body mass index; CVD, cardiovascular disease; eGFR, estimated glomerular filtration ratio; HDL-C, high-density lipoprotein cholesterol; hsCRP, high-sensitivity C-reactive protein;IQR, interquartile range; LDL-C, low-density lipoprotein cholesterol; TyG, triglyceride-glucose.

We first evaluated the association of BMI (both as a linear term and classification) and TyG index (both linearly and in quartiles) with CVD using Cox proportional hazard models. Additionally, we explored linear trends through entering the median value of each BMI group or TyG index quartile to test association across the various BMI groups or TyG index quartiles. We then assessed the association of BMI with mediators using linear models. All models were adjusted for age, gender, marital status, residence, education level, smoking status, and drinking status. Subsequently, we applied 4-way decomposition causal mediation techniques to estimate the controlled direct association (CDA), reference interaction (INTref), mediated interaction (INTmed), and pure indirect association (PIA) individually (29). Utilizing the framework depicted in **Figure 1** (30).

To evaluate the indirect and direct association between BMI and CVD events, we utilized VanderWeele’s two-stage regression method for time-to-event data (29, 31). This approach involves fitting two regression models: one for the mediator (TyG index) and another for the outcome (CVD). The outcome (CVD) was modeled using a Cox model, while the mediator (TyG index) was modeled using a linear model. We also conducted similar mediation analyses using BMI categories, treating the TyG index as a linear indicator due to its confirmed linear association with CVD risk. All models were adjusted for age, gender, marital status, residence, education level, smoking status, and drinking status. Subsequently, we used the model parameters from these models to calculated the CDA, INTref, INTmed, and PIA, estimating the proportions of the total excess association attributable to each component according to VanderWeele’s derivations (29). The 95% CIs for estimates and proportion mediated were calculated by delta method (32).

We implemented several sensitivity analyses to assess the robustness. Initially, we conducted mediation analyses according to gender. Subsequently, we assessed our results using the complete dataset (6884 participants). Finally, we repeated the mediation analysis excluding participants with a BMI <18.5 kg/m² (6723 participants). We considered two-sided P < 0.05 as statistically significant. All analyses were carried out using R statistical software version 4.3.0 (R Foundation), and mediation analysis was performed using the CMAverse package developed by Baoyi Shi, Christine Choirat, and Linda Valeri (https://bs1125.github.io/CMAverse/index.html).

## Results

### Baseline characteristics

There were 17 708 participants at baseline, we excluded 777 participants below 45 years, 2 650 with baseline heart disease or stroke, 5 622 had no blood samples, 1 409 had incomplete TyG index or BMI, and 17 with extreme BMI values. Finally, 7 233 participants were included for analysis. Baseline characteristics between included and excluded participants is shown in **Supplementary Table S1**.

Of the 7 233 participants, the mean (SD) age at baseline was 58.93 (9.33) years; 3 415 (47.2%) were men. Participants’ characteristics are presented in **Table 1**. At baseline, 2 088 (28.9%) participants had overweight and 755 (10.4%) had obsity, the mean (SD) TyG index was 8.65 (0.65).

**Table 1:** Baseline characteristics participants stratified by quartiles of the TyG index.

Characteristic	Overall	TyG index ^a^				P value ^b^
		Quartile 1 [5.18, 8.20]	Quartile 2 (8.20, 8.57]	Quartile 3 (8.57, 9.00]	Quartile 4 (9.00, 13.00]	
No.	7233	1810	1807	1808	1808	
Age, years	58.93 ± 9.33	58.95 ± 9.66	58.86 ± 9.37	59.44 ± 9.39	58.49 ± 8.87	0.020
Gender						<0.001
Male	3415 (47.2%)	1004 (55.5%)	872 (48.3%)	770 (42.6%)	769 (42.5%)	
Female	3818 (52.8%)	806 (44.5%)	935 (51.7%)	1038 (57.4%)	1039 (57.5%)	
Marital status						0.057
Marred	6045 (83.6%)	1509 (83.4%)	1522 (84.2%)	1478 (81.7%)	1536 (85.0%)	
Other	1188 (16.4%)	301 (16.6%)	285 (15.8%)	330 (18.3%)	272 (15.0%)	
Residence						<0.001
Urban	2493 (34.5%)	514 (28.4%)	593 (32.8%)	637 (35.2%)	749 (41.4%)	
Rural	4740 (65.5%)	1296 (71.6%)	1214 (67.2%)	1171 (64.8%)	1059 (58.6%)	
Education level						0.003
No formal education	2200 (30.4%)	538 (29.7%)	534 (29.6%)	592 (32.7%)	536 (29.6%)	
Primary school	2912 (40.3%)	754 (41.7%)	751 (41.6%)	701 (38.8%)	706 (39.0%)	
Middle or high school	1915 (26.5%)	482 (26.6%)	466 (25.8%)	474 (26.2%)	493 (27.3%)	
College or above	206 (2.8%)	36 (2.0%)	56 (3.1%)	41 (2.3%)	73 (4.0%)	
Smoking status ^c^						<0.001
Never	4381 (60.6%)	1001 (55.3%)	1080 (59.8%)	1131 (62.6%)	1169 (64.7%)	
Former	589 (8.1%)	151 (8.3%)	143 (7.9%)	146 (8.1%)	149 (8.2%)	
Current	2243 (31.0%)	652 (36.0%)	580 (32.1%)	523 (28.9%)	488 (27.0%)	
Drinking status ^c^						<0.001
Never	4168 (57.6%)	963 (53.2%)	1022 (56.6%)	1113 (61.6%)	1070 (59.2%)	
Former	583 (8.1%)	138 (7.6%)	157 (8.7%)	155 (8.6%)	133 (7.4%)	
Current	2478 (34.3%)	709 (39.2%)	627 (34.7%)	538 (29.8%)	604 (33.4%)	
Body mass index, kg/m^2^						<0.001
Under and normal (<24.0)	4390 (60.7%)	1396 (77.1%)	1212 (67.1%)	1020 (56.4%)	762 (42.1%)	
Overweight (24.0-27.9)	2088 (28.9%)	342 (18.9%)	463 (25.6%)	584 (32.3%)	699 (38.7%)	
Obesity (≥28.0)	755 (10.4%)	72 (4.0%)	132 (7.3%)	204 (11.3%)	347 (19.2%)	
History of comorbidities						
Hypertension ^c^	1660 (23.0%)	281 (15.5%)	330 (18.3%)	464 (25.7%)	585 (32.4%)	<0.001
Diabetes ^c^	373 (5.2%)	23 (1.3%)	49 (2.7%)	83 (4.6%)	218 (12.1%)	<0.001
Dyslipidemia ^c^	557 (7.7%)	62 (3.4%)	111 (6.1%)	143 (7.9%)	241 (13.3%)	<0.001
Kidney disease ^c^	362 (5.0%)	94 (5.2%)	100 (5.5%)	85 (4.7%)	83 (4.6%)	0.530
History of medication use						
Hypertension medications ^c^	1198 (16.6%)	181 (10.0%)	231 (12.8%)	340 (18.8%)	446 (24.7%)	<0.001
Diabetes medications ^c^	231 (3.2%)	12 (0.7%)	28 (1.5%)	46 (2.5%)	145 (8.0%)	<0.001
Dyslipidemia medications ^c^	265 (3.7%)	25 (1.4%)	51 (2.8%)	67 (3.7%)	122 (6.7%)	<0.001
Systole blood pressure, mmHg ^c^	128.82 ± 21.02	125.04 ± 20.58	127.05 ± 20.36	130.53 ± 21.97	132.68 ± 20.31	<0.001
Diastolic blood pressure, mmHg ^c^	75.02 ± 12.05	72.78 ± 11.81	74.18 ± 11.70	75.82 ± 12.41	77.31 ± 11.81	<0.001
Total cholesterol, mg/dl	194.31 ± 38.43	179.44 ± 33.30	190.32 ± 33.76	198.05 ± 36.72	209.46 ± 42.79	<0.001
HDL-C, mg/dl	51.82 ± 15.26	60.74 ± 15.17	55.22 ± 13.87	49.80 ± 13.02	41.50 ± 11.73	<0.001
LDL-C, mg/dl ^c^	117.47 ± 34.60	108.88 ± 29.31	119.19 ± 30.72	124.77 ± 34.00	117.05 ± 41.28	<0.001
Glycated hemoglobin, % ^c^	5.28 ± 0.81	5.08 ± 0.42	5.15 ± 0.50	5.21 ± 0.58	5.67 ± 1.28	<0.001
Median hsCRP (IQR), mg/l	1.01 (0.54, 2.14)	0.80 (0.46, 1.81)	0.89 (0.50, 1.91)	1.05 (0.59, 2.14)	1.30 (0.70, 2.59)	<0.001
eGFR, ml/min/1.73m^2^ ^c^	76.79 ± 43.61	75.14 ± 39.49	75.79 ± 57.26	76.42 ± 35.20	79.81 ± 38.99	0.002

Data are presented as mean ± SD or n(%), unless otherwise specified.

eGFR, estimated glomerular filtration ratio; HDL-C, high-density lipoprotein cholesterol; hsCRP, high-sensitivity C-reactive protein; IQR, interquartile range; LDL-C, low-density lipoprotein cholesterol; TyG, triglyceride-glucose.

a TyG index was calculated as ln (triglycerides [milligrams per deciliter] × fasting blood glucose [milligrams per deciliter]/2).

b P value was based on χ^2^, analysis of variance test or Kruskal-Wallis rank sum test where appropriate.

c Missing data: 20 for smoking status, 4 for drinking status, 32 for hypertension, 62 for diabetes, 145 for dyslipidemia, 24 for kidney disease, 33 for history of medication use for hypertension, 63 for history of medication use for diabetes, 147 for history of medication use for dyslipidemia, 70 for systole blood pressure, 71 for diastolic blood pressure, 13 for LDL-C, 61 for HbA1c, and 2 for eGFR.

### Risk of CVD by TyG index or BMI

Between 2011 and 2018, 1411 participants experienced CVD events, including 1 077 heart attacks and 464 strokes, a 19.5% incidence rate. In **Table 2**, we show how BMI and TyG index are related to CVD events after adjusting for potential confounders (model 2), by comparing to under and normal weight, obesity was associated with a 90.0% increased risk of incident CVD (CVD: adjusted HR, 1.90; 95% CI, 1.63–2.22; stroke: adjusted HR, 2.13; 95% CI, 1.63–2.78; heart disease: adjusted HR, 1.85; 95% CI, 1.55–2.20). When modeling the TyG index as quartiles, by comparing quartile 4 with quartile 1, the adjusted HRs were 1.35 (95% CI, 1.16–1.57) for incident CVD, 2.17 (95% CI, 1.63–2.88) for stroke, and 1.14 (95% CI, 0.96–1.35) for heart disease. BMI and CVD risk are linearly associated and positive (for trend, P <0.001 for CVD, stroke, and heart disease), as well as, the TyG indx (for trend, P <0.001 for CVD and stroke, P = 0.131 for heart disease).

**Table 2:** Risk of cardiovascular disease by TyG index or body mass index.

Outcome	No. of event/total	Model 1 ^a^			Model 2 ^b^		
		HR (95% CI)	P value	P for trend ^c^	HR (95% CI)	P value	P for trend ^c^
Cardiovascular disease							
Body mass index, kg/m^2^				<0.001			<0.001
Under and normal (<24.0)	740/4390	1.00 [Reference]			1.00 [Reference]		
Overweight (24.0–27.9)	448/2088	1.30 (1.15–1.46)	<0.001		1.28 (1.14–1.44)	<0.001	
Obesity (≥28.0)	223/755	1.93 (1.66–2.25)	<0.001		1.90 (1.63–2.22)	<0.001	
Body mass index continuous ^e^	1411/7233	1.23 (1.17–1.29)	<0.001		1.23 (1.17–1.29)	<0.001	
Quartiles of the TyG index ^d^				<0.001			<0.001
Quartile 1 [5.18, 8.2.0]	289/1810	1.00 [Reference]			1.00 [Reference]		
Quartile 2 (8.20, 8.57]	340/1807	1.16 (0.99–1.36)	0.065		1.14 (0.98–1.34)	0.100	
Quartile 3 (8.57, 9.00]	385/1808	1.31 (1.12–1.53)	0.001		1.29 (1.10–1.50)	0.001	
Quartile 4 (9.00, 13.00]	397/1808	1.38 (1.18–1.61)	<0.001		1.35 (1.16–1.57)	<0.001	
TyG index continuous ^e^	1411/7233	1.14 (1.08–1.20)	<0.001		1.13 (1.07–1.19)	<0.001	
Stroke				<0.001			<0.001
Body mass index, kg/m^2^							
Under and normal (<24.0)	238/4390	1.00 [Reference]			1.00 [Reference]		
Overweight (24.0–27.9)	150/2088	1.39 (1.13–1.71)	0.002		1.42 (1.15–1.75)	0.001	
Obesity (≥28.0)	76/755	2.12 (1.63–2.76)	<0.001		2.13 (1.63–2.78)	<0.001	
Body mass index continuous ^e^	464/7233	1.29 (1.19–1.40)	<0.001		1.29 (1.19–1.40)	<0.001	
Quartiles of the TyG index ^d^				<0.001			<0.001
Quartile 1 [5.18, 8.2.0]	73/1810	1.00 [Reference]			1.00 [Reference]		
Quartile 2 (8.20, 8.57]	101/1807	1.40 (1.04–1.90)	0.028		1.40 (1.03–1.89)	0.031	
Quartile 3 (8.57, 9.00]	141/1808	1.99 (1.49–2.64)	<0.001		1.96 (1.48–2.61)	<0.001	
Quartile 4 (9.00, 13.00]	149/1808	2.17 (1.64–2.88)	<0.001		2.17 (1.63–2.88)	<0.001	
TyG index continuous e	464/7233	1.31 (1.21–1.42)	<0.001		1.31 (1.21–1.42)	<0.001	
Heart disease							
Body mass index, kg/m^2^				<0.001			<0.001
Under and normal (<24.0)	559/4390	1.00 [Reference]			1.00 [Reference]		
Overweight (24.0–27.9)	345/2088	1.29 (1.13–1.48)	<0.001		1.26 (1.10–1.45)	0.001	
Obesity (≥28.0)	173/755	1.90 (1.60–2.26)	<0.001		1.85 (1.55–2.20)	<0.001	
Body mass index continuous ^e^	1077/7233	1.22 (1.16–1.29)	<0.001		1.21 (1.14–1.28)	<0.001	
Quartiles of the TyG index ^d^				0.059			0.131
Quartile 1 [5.18, 8.2.0]	239/1810	1.00 [Reference]			1.00 [Reference]		
Quartile 2 (8.20, 8.57]	265/1807	1.08 (0.91–1.29)	0.396		1.06 (0.89–1.26)	0.525	
Quartile 3 (8.57, 9.00]	282/1808	1.13 (0.95–1.34)	0.180		1.10 (0.93–1.31)	0.266	
Quartile 4 (9.00, 13.00]	291/1808	1.18 (0.99–1.40)	0.063		1.14 (0.96–1.35)	0.143	
TyG index continuous ^e^	1077/7233	1.07 (1.01–1.14)	0.016		1.06 (1.00–1.13)	0.044	

HR, hazard ratio; CI, confidence interval; TyG, triglyceride-glucose.

a Adjusted for age and gender.

b Adjusted for age, gender, marital status, residence, education level, smoking status, and drinking status.

c Tests for linear trend were done by modeling the median value of each group to test ordered relations across groups of body mass index or TyG index.

d TyG index was calculated as ln (triglycerides [milligrams per deciliter] × fasting blood glucose [milligrams per deciliter]/2).

e HRs given per 1-SD increase.

### Association of BMI and TyG index

**Table 3** shows the associations of BMI with TyG index. After adjusting for potential confounders (in model 2), compared with participants with underweight and normal weight, participants with overweight and obesity had higher TyG index (overweight: adjusted β, 0.28; 95% CI, 0.25– 0.32; obesity: adjusted β, 0.47; 95% CI, 0.42–0.52).

**Table 3:** Association between body mass index and TyG index.

Body mass index, kg/m^2^	No. of total	Model 1 ^a^		Model 2 ^b^	
		β (95% CI)	P value	β (95% CI)	P value
Under and normal (<24.0)	4390	0.00 [Reference]		0.00 [Reference]	
Overweight (24.0–27.9)	2088	0.30 (0.26–0.33)	<0.001	0.28 (0.25–0.32)	<0.001
Obesity (≥28.0)	755	0.48 (0.43–0.53)	<0.001	0.47 (0.42–0.52)	<0.001

CI, confidence interval.

a Adjusted for age and gender.

b Adjusted for age, gender, marital status, residence, education level, smoking status, and drinking status.

### Mediation and interaction analysis

**Table 4** show the findings from 4-way decomposition model. Analysis by BMI categories yielded the adjusted HR for the total association of BMI with incident CVD was 1.28 for overweight *vs* the reference normal weight (CVD: adjusted HR, 1.28; 95% CI, 1.14–1.45; stroke: adjusted HR, 1.41; 95% CI, 1.15–1.74; heart disease: adjusted HR, 1.26; 95% CI, 1.10–1.45), which increased to 1.91 for the obesity group (CVD: adjusted HR, 1.91; 95% CI, 1.63–2.23; stroke: adjusted HR, 2.13; 95% CI, 1.63–2.78; heart disease: adjusted HR, 1.85; 95% CI, 1.55–2.21). The 4 components method show that when using TyG index as a mediator, There was no evidence that BMI interacted with TyG via INTref or INTmed, and the majority of the association was direct, with the remainder being purely indirect, the proportions mediated were 18.1% for overweight (CVD: 18.1%; 95% CI, 2.2%–34.0%; stroke: 30.3%; 95% CI, 4.1%–56.5%), and 9.5% for obesity (CVD: 9.5%; 95% CI, 2.2%–16.7%; stroke: 19.1%; 95% CI, 5.6%–32.7%). Notably, for heart disease, virtually all of the association was direct, no evidence of mediation and interaction.

**Table 4:** Decomposition of the association of body mass index with incident cardiovascular disease including mediation and interaction associations by TyG index using causal mediation analysis^a^.

Association component	Overweight (Ref. Under and normal weight)				Obesity (Ref. Under and normal weight)			
	HR (95% CI)	P value	Percentage of excess association (95% CI)	P value	HR (95% CI)	P value	Percentage of excess association (95% CI)	P value
Cardiovascular disease								
Total association	1.28 (1.14 to 1.45)	<0.001	100.0		1.91 (1.63 to 2.23)	<0.001	100.0	
Controlled direct association	1.28 (1.13 to 1.45)	<0.001	98.6 (27.7 to 169.6)	0.006	1.76 (1.47 to 2.10)	<0.001	83.4 (69.6 to 97.3)	<0.001
Reference interaction ^b^	0.00 (-0.10 to 0.10)	0.987	0.3 (-72.2 to 72.8)	0.993	-0.00 (-0.20 to 0.19)	0.992	-0.1 (-18.3 to 18.1)	0.991
Mediated interaction ^b^	-0.05 (-0.11 to 0.01)	0.129	-17.0 (-40.8 to 6.8)	0.160	0.07 (-0.10 to 0.23)	0.445	7.2 (-11.1 to 25.5)	0.440
Pure indirect association	1.05 (1.02 to 1.09)	0.004	18.1 (2.2 to 34.0)	0.025	1.09 (1.03 to 1.15)	0.004	9.5 (2.2 to 16.7)	0.011
Stroke								
Total association	1.41 (1.15 to 1.74)	0.001	100.0		2.13 (1.63 to 2.78)	<0.001	100.0	
Controlled direct association	1.35 (1.08 to 1.69)	0.008	84.0 (23.7 to 144.4)	0.006	1.72 (1.24 to 2.38)	0.001	62.3 (46.3 to 78.3)	<0.001
Reference interaction ^b^	-0.02 (-0.20 to 0.17)	0.861	-4.0 (-74.9 to 67.0)	0.913	0.02 (-0.31 to 0.34)	0.907	1.7 (-22.9 to 26.4)	0.892
Mediated interaction ^b^	-0.04 (-0.15 to 0.06)	0.430	-10.4 (-38.3 to 17.5)	0.465	0.19 (-0.09 to 0.47)	0.177	16.9 (-6.0 to 39.7)	0.148
Pure indirect association	1.13 (1.07 to 1.19)	<0.001	30.3 (4.1 to 56.5)	0.023	1.22 (1.11 to 1.33)	<0.001	19.1 (5.6 to 32.7)	0.006
Heart disease								
Total association	1.26 (1.10 to 1.45)	0.001	100.0		1.85 (1.55 to 2.21)	<0.001	100.0	
Controlled direct association	1.27 (1.10 to 1.47)	0.001	103.5 (10.2 to 196.8)	0.030	1.82 (1.49 to 2.23)	<0.001	96.6 (78.5 to 114.6)	<0.001
Reference interaction ^a^	0.00 (-0.12 to 0.12)	0.948	1.5 (-90.7 to 93.7)	0.974	-0.00 (-0.24 to 0.24)	0.997	-0.1 (-22.4 to 22.3)	0.996
Mediated interaction ^a^	-0.03 (-0.10 to 0.04)	0.428	-11.0 (-38.8 to 16.8)	0.439	0.00 (-0.20 to 0.21)	0.971	0.4 (-23.4 to 24.3)	0.971
Pure indirect association	1.02 (0.98 to 1.06)	0.440	6.0 (-9.8 to 21.7)	0.457	1.03 (0.96 to 1.10)	0.440	3.1 (-4.9 to 11.0)	0.450

HR, hazard ratio; CI, confidence interval; TyG, triglyceride-glucose.

a Decomposition of total associations into controlled direct association (CDA), reference interaction (INTref), mediated interaction (INTmed), and pure indirect association (PIA) was done according to the 4-way decomposition causal mediation analysis method proposed by VanderWeele. CIs were calculated according to the delta method procedure. All models were adjusted for age, gender, marital status, residence, education level, smoking status, and drinking status as depicted in the directed acyclic graph (DAG).

b INTref and INTmed are the estimation of additive excess relative risk due to interaction using HRs.

### Subgroup and sensitivity analysis

Subgroup analysis among women (**Supplementary Table S2**), the proportions mediated of TyG index between BMI and CVD were increased (overweight, 37.5%; obesity, 12.3%), while the proportions mediated decreased (overweight, 6.2%; obesity, 4.6%) among men (**Supplementary Table S3**). Similar trends were observed in the complete data analysis (**Supplementary Table S4**). Moreover, the results remained consistent even after excluding participants with a BMI <18.5 kg/m^2^ (**Supplementary Table S5**).

## Discussion

In this large cohort study, we found that the TyG index independently raised the risk of CVD. Additionally, a minor portion of the BMI-CVD association was mediated by the TyG index. Epidemiological studies consistently show a positive correlation between higher BMI and subsequent CVD risk (33–37). Our study findings align with these conclusions, revealing that an increase in BMI by per 1-SD increased the risk of CVD by 23%. Moreover, stratifying participants by BMI categories revealed a 28% increased CVD risk among overweight individuals and a nearly twofold elevation (HR, 1.90) among those with obesity compared to the baseline population of normal weight. Notably, individuals classified as overweight or obese exhibit a higher propensity for developing insulin resistance, signaling early signs of disrupted glucose metabolism (38, 39). Epidemiological study have showed a direct correlation between insulin resistance and CVD, which persists independently of diabetes and is aggravated when obesity (40). Thus, BMI and CVD risk may be mediated by insulin resistance.

Our study furnishes empirical evidence substantiating the biologically conceivable conjecture that insulin resistance serves as a pivotal intermediary in the linkage between obesity and CVD. We determined that the TyG index accounted for 18.1% of the mediating proportion in cases of overweight and 9.5% in instances of general obesity. Notably, insulin resistance frequently coexists with an array of traditional risk factors including dyslipidemia, glucose dysregulation, and hypertension, all of which have been corroborated in prior research as mediators in the causal pathway between obesity and CVD (41, 42). A retrospective cohort analysis including 6 078 participants aged 60 years and older elucidated that the TyG index served as a mediator in the relationship between BMI and CVD events. Previous studies have not firmly established insulin resistance’s role in BMI and CVD. However, a study involving 6078 participants aged ≥60 years showed that BMI and CVD events were the mediated by TyG index. But, the study did not furnish information regarding the proportion mediated (43). Another prospective cohort study of 94 136 participants in which most were coal miners revealed that TyG index was a mediator in the relationship between BMI and CVD events (proportion mediated: 47.81% for overweight, 37.94% for obesity) (20). Limitations include population heterogeneity, absence of generalizable, which results in a higher proportion of TyG mediation compared to our results. In contrast, our prospective analysis centered on the general population and employed a novel method to calculate the mediated proportion of TyG index. Collectively, our results, along with previous studies, suggest that controlling the TyG index may help mitigate the effects of BMI on CVD. However, this effect may not be pronounced in the Chinese general population.

The deleterious impact of BMI on CVD susceptibility is well-documented. The underlying pathophysiological mechanisms potentially involve several pathways. Adipose tissue expansion instigates heightened basal lipolysis, liberating free fatty acids (FFA), interleukins, and cytokines. These biochemical mediators contribute to cardiac dysfunction by expediting atherosclerotic progression and modulating factors implicated in inflammation, endothelial dysfunction, and coagulation abnormalities (44). Elevated FFA levels attributable to obesity precipitate insulin resistance, exacerbating impaired insulin signaling and attenuating insulin-mediated glucose uptake in skeletal muscle while augmenting hepatic glucose output (45). Moreover, a state of positive energy balance engenders adipocyte hypertrophy and ectopic fat deposition, fostering metabolic perturbations such as insulin resistance and beta-cell dysfunction (46). Additionally, the pro-inflammatory milieu associated with obesity potentiates lipolytic processes and hepatic triglyceride synthesis, exacerbating hyperlipidemia through heightened fatty acid esterification (13). Notably, insulin resistance constitutes a pivotal nexus in the interplay between obesity and CVD risk. Consequently, the TyG index emerges as a plausible intermediary linking obesity with heightened CVD susceptibility.

Our findings are notable as they stem from a comprehensive, representative cohort of the Chinese general population, with a prolonged follow-up period. This extended duration is crucial for meaningfully exploring longitudinal associations, especially those concerning obesity and CVD. Additionally, we applied a counterfactual framework to analyze mediation in an innovative way, our implementation of the 4-way decomposition approach enabled the simultaneous examination of the TyG index’s role as both modifiers and mediators.

However, our study also has limitations. First, we used BMI to ascertain overweight and obesity, while widely employed and easily calculable, offers a suboptimal estimate of fat mass proportion and distribution. There was a lack of alternative metrics, such as waist circumference (47), waist-to-hip ratio (47, 48), or body fat composition analysis (49), that could be used to quantify visceral fat more accurately. Second, the reliance on self-reporting for CVD diagnosis introduces a methodological challenge. While the CHARLS dataset lacks medical records, preventing the validation of self-reported CVD incidents, it’s important to acknowledge that other large-scale studies, like the English Longitudinal Study of Ageing, have demonstrated notable agreement between self-reported CVDs and medical records (50). Third, although we cannot definitively rule out the possibility of unmeasured confounding, the observed effect sizes’ magnitude makes it improbable for unmeasured confounding to entirely elucidate our observed associations. Four, the concurrent measurement of BMI and the TyG index at baseline does not guarantee temporality between exposure and mediator, introducing the potential for reverse causality. However, there exists sufficient biological rationale and explanation for BMI influencing insulin resistance (51–53). Last, due to the considerable sample size and associated costs, data on insulin resistance were not collected, preventing the use of homeostasis model assessment of insulin resistance (HOMA-IR) for reflecting insulin resistance, necessitating further investigations.

## Conclusions

Our results indicate that the TyG index is valuable for identifying individuals prone to CVD development. Additionally, it acts as a minor mediator in the association between BMI and CVD within our general population cohort. Consequently, further exploration of the pathways connecting BMI to CVD is essential for comprehending disease origins and pinpointing populations that could gain the most from strategies aimed at reducing BMI.

## Data Availability

Publicly available datasets were analyzed in this study. This data can be found here: https://charls.pku.edu.cn/en/.
