# Supplementary material for: Triglyceride-glucose index as a mediator of body mass index and cardiovascular disease in middle-aged and older Chinese adults: a nationally representative longitudinal cohort study
Source: Front Endocrinol (Lausanne). 2024 Dec 23;15:1431087. doi: 10.3389/fendo.2024.1431087 (PMC11700812; doi:10.3389/fendo.2024.1431087)
Supplement: Supplementary file 1 [file Table1.docx]

Table S1: Baseline characteristics between participants included and not included

| Characteristic | Exclude (n=10475) | Include (n=7233) | P value ^a^ |
| --- | --- | --- | --- |
| Age, years |  |  | <0.001 |
| Mean±SD | 58.19±10.71 | 58.93±9.33 |  |
| Missing | 175 (1.7%) | 0 (0.0%) |  |
| Gender |  |  | 0.139 |
| Male | 5063 (48.3%) | 3415 (47.2%) |  |
| Female | 5410 (51.6%) | 3818 (52.8%) |  |
| Missing | 2 (0.0%) | 0 (0.0%) |  |
| Marital status |  |  | <0.001 |
| Marred | 8125 (77.6%) | 6045 (83.6%) |  |
| Other | 2338 (22.3%) | 1188 (16.4%) |  |
| Missing | 12 (0.1%) | 0 (0.0%) |  |
| Residence |  |  | <0.001 |
| Urban | 4678 (44.7%) | 2493 (34.5%) |  |
| Rural | 5797 (55.3%) | 4740 (65.5%) |  |
| Education level |  |  | <0.001 |
| No formal education | 2651 (25.3%) | 2200 (30.4%) |  |
| Primary school | 4008 (38.3%) | 2912 (40.3%) |  |
| Middle or high school | 3188 (30.4%) | 1915 (26.5%) |  |
| College or above | 612 (5.8%) | 206 (2.8%) |  |
| Missing | 16 (0.2%) | 0 (0.0%) |  |
| Smoking status |  |  | <0.001 |
| Never | 6238 (59.6%) | 4381 (60.6%) |  |
| Former | 828 (7.9%) | 589 (8.1%) |  |
| Current | 2628 (25.1%) | 2243 (31.0%) |  |
| Missing | 781 (7.5%) | 20 (0.3%) |  |
| Drinking status |  |  | 0.004 |
| Never | 6165 (58.9%) | 4168 (57.6%) |  |
| Former | 860 (8.2%) | 583 (8.1%) |  |
| Current | 3289 (31.4%) | 2478 (34.3%) |  |
| Missing | 161 (1.5%) | 4 (0.1%) |  |
| Body mass index, kg/m^2^ |  |  | 0.001 |
| <24.0 | 3753 (35.8%) | 4390 (60.7%) |  |
| 24.0-27.9 | 1854 (17.7%) | 2088 (28.9%) |  |
| ≥28.0 | 791 (7.6%) | 755 (10.4%) |  |
| Missing | 4077 (38.9%) | 0 (0.0%) |  |
| Hypertension |  |  | <0.001 |
| No | 7394 (70.6%) | 5541 (76.6%) |  |
| Yes | 2875 (27.4%) | 1660 (23.0%) |  |
| Missing | 206 (2.0%) | 32 (0.4%) |  |
| Diabetes |  |  | <0.001 |
| No | 9548 (91.2%) | 6798 (94.0%) |  |
| Yes | 689 (6.6%) | 373 (5.2%) |  |
| Missing | 238 (2.3%) | 62 (0.9%) |  |
| Dyslipidemia |  |  | <0.001 |
| No | 8986 (85.8%) | 6531 (90.3%) |  |
| Yes | 1139 (10.9%) | 557 (7.7%) |  |
| Missing | 350 (3.3%) | 145 (2.0%) |  |
| Kidney disease |  |  | 0.005 |
| No | 9626 (91.9%) | 6847 (94.7%) |  |
| Yes | 617 (5.9%) | 362 (5.0%) |  |
| Missing | 232 (2.2%) | 24 (0.3%) |  |
| History of medication use for hypertension |  |  | <0.001 |
| No | 8044 (76.8%) | 6002 (83.0%) |  |
| Yes | 2216 (21.2%) | 1198 (16.6%) |  |
| Missing | 215 (2.1%) | 33 (0.5%) |  |
| History of medication use for diabetes |  |  | <0.001 |
| No | 9753 (93.1%) | 6939 (95.9%) |  |
| Yes | 481 (4.6%) | 231 (3.2%) |  |
| Missing | 241 (2.3%) | 63 (0.9%) |  |
| History of medication use for dyslipidemia |  |  | <0.001 |
| No | 9515 (90.8%) | 6821 (94.3%) |  |
| Yes | 600 (5.7%) | 265 (3.7%) |  |
| Missing | 360 (3.4%) | 147 (2.0%) |  |
| Systole blood pressure, mmHg |  |  | <0.001 |
| Mean±SD | 130.42±22.10 | 128.82±21.02 |  |
| Missing | 3898 (37.2%) | 70 (1.0%) |  |
| Diastolic blood pressure, mmHg |  |  | <0.001 |
| Mean±SD | 75.99±12.45 | 75.02±12.05 |  |
| Missing | 3898 (37.2%) | 71 (1.0%) |  |
| Total cholesterol, mg/dl |  |  | <0.001 |
| Mean±SD | 190.76±39.55 | 194.31±38.43 |  |
| Missing | 6053 (57.8%) | 0 (0.0%) |  |
| HDL-C, mg/dl |  |  | <0.001 |
| Mean±SD | 49.25±15.31 | 51.82±15.26 |  |
| Missing | 6045 (57.7%) | 0 (0.0%) |  |
| LDL-C, mg/dl |  |  | <0.001 |
| Mean±SD | 113.59±35.29 | 117.47±34.60 |  |
| Missing | 6053 (57.8%) | 13 (0.2%) |  |
| Glycated hemoglobin, % |  |  | 0.037 |
| Mean±SD | 5.24±0.85 | 5.28±0.81 |  |
| Missing | 5941 (56.7%) | 61 (0.8%) |  |
| Median hsCRP (IQR), mg/l |  |  | 0.006 |
| Median (IQR) | 1.09 (0.56, 2.32) | 1.01 (0.54, 2.14) |  |
| Missing | 6044 (57.7%) | 0 (0.0%) |  |

Data are presented as mean±SD or n(%), unless otherwise specified.

Abbreviations: HDL-C, high-density lipoprotein cholesterol; hsCRP, high-sensitivity C-reactive protein; IQR, interquartile range; LDL-C, low-density lipoprotein cholesterol.

^a^ P value was based on χ^2^ , analysis of variance test or Kruskal-Wallis rank sum test where appropriate.

Table S2: Decomposition of the association of body mass index with incident cardiovascular disease including mediation and interaction associations by TyG index using causal mediation analysis in subpopulations of 3 818 female participants ^a^

| Association component | Overweight (Ref. Under and normal weight) | | | |  | Obesity (Ref. Under and normal weight) | | | |
| --- | --- | --- | --- | --- | --- | --- | --- | --- | --- |
|  | HR (95% CI) | P value | Percentage of excess  association (95% CI) | P value |  | HR (95% CI) | P value | Percentage of excess  association (95% CI) | P value |
| Cardiovascular disease |  |  |  |  |  |  |  |  |  |
| Total association | 1.14 (0.97 to 1.33) | 0.108 | 100.0 |  |  | 1.72 (1.42 to 2.08) | <0.001 | 100.0 |  |
| Controlled direct association | 1.14 (0.97 to 1.34) | 0.099 | 105.5 (-246.0 to 457.0) | 0.556 |  | 1.66 (1.34 to 2.05) | <0.001 | 91.7 (67.3 to 116.1) | <0.001 |
| Reference interaction ^b^ | 0.01 (-0.12 to 0.13) | 0.932 | 3.8 (-338.6 to 346.3) | 0.983 |  | -0.00 (-0.23 to 0.22) | 0.979 | -0.4 (-29.7 to 28.9) | 0.977 |
| Mediated interaction ^b^ | -0.06 (-0.13 to -0.00) | 0.050 | -46.8 (-121.8 to 28.3) | 0.222 |  | -0.03 (-0.21 to 0.16) | 0.787 | -3.6 (-29.8 to 22.7) | 0.789 |
| Pure indirect association | 1.05 (1.01 to 1.09) | 0.008 | 37.5 (-18.9 to 93.9) | 0.193 |  | 1.09 (1.02 to 1.16) | 0.007 | 12.3 (1.4 to 23.3) | 0.027 |
| Stroke |  |  |  |  |  |  |  |  |  |
| Total association | 1.23 (0.91 to 1.65) | 0.172 | 100.0 |  |  | 1.94 (1.36 to 2.76) | <0.001 | 100.0 |  |
| Controlled direct association | 1.18 (0.87 to 1.62) | 0.289 | 79.7 (-163.4 to 322.9) | 0.520 |  | 1.59 (1.04 to 2.41) | 0.031 | 62.1 (33.2 to 90.9) | <0.001 |
| Reference interaction ^b^ | -0.01 (-0.24 to 0.22) | 0.931 | -4.4 (-280.8 to 272.1) | 0.975 |  | 0.02 (-0.36 to 0.41) | 0.903 | 2.5 (-36.7 to 41.8) | 0.899 |
| Mediated interaction ^b^ | -0.03 (-0.15 to 0.08) | 0.585 | -14.1 (-72.0 to 43.7) | 0.632 |  | 0.18 (-0.17 to 0.52) | 0.321 | 18.8 (-15.3 to 52.9) | 0.280 |
| Pure indirect association | 1.09 (1.02 to 1.16) | 0.013 | 38.8 (-30.1 to 107.6) | 0.270 |  | 1.16 (1.03 to 1.29) | 0.012 | 16.6 (-1.7 to 34.8) | 0.075 |
| Heart disease |  |  |  |  |  |  |  |  |  |
| Total association | 1.17 (0.98 to 1.39) | 0.077 | 100.0 |  |  | 1.75 (1.42 to 2.16) | <0.001 | 100.0 |  |
| Controlled direct association | 1.18 (0.99 to 1.41) | 0.066 | 106.9 (-155.2 to 368.9) | 0.424 |  | 1.75 (1.39 to 2.22) | <0.001 | 100.5 (74.9 to 126.1) | <0.001 |
| Reference interaction ^a^ | 0.01 (-0.13 to 0.15) | 0.899 | 5.3 (-246.1 to 256.7) | 0.967 |  | 0.00 (-0.27 to 0.28) | 0.975 | 0.6 (-29.7 to 30.8) | 0.970 |
| Mediated interaction ^a^ | -0.06 (-0.13 to 0.01) | 0.118 | -34.0 (-91.1 to 23.1) | 0.243 |  | -0.07 (-0.29 to 0.15) | 0.517 | -9.6 (-38.8 to 19.7) | 0.522 |
| Pure indirect association | 1.04 (1.00 to 1.08) | 0.084 | 21.9 (-14.5 to 58.2) | 0.239 |  | 1.06 (0.99 to 1.14) | 0.082 | 8.5 (-2.2 to 19.2) | 0.121 |

Abbreviations: HR, hazard ratio; CI, confidence interval; TyG, triglyceride-glucose.

^a^ Decomposition of total associations into controlled direct association (CDA), reference interaction (INTref), mediated interaction (INTmed), and pure indirect association (PIA) was done according to the 4-way decomposition causal mediation analysis method proposed by VanderWeele. CIs were calculated according to the delta method procedure. All models were adjusted for age, gender, marital status, residence, education level, smoking status, and drinking status as depicted in the directed acyclic graph (DAG).

^b^ INTref and INTmed are the estimation of additive excess relative risk due to interaction using HRs.

Table S3: Decomposition of the association of body mass index with incident cardiovascular disease including mediation and interaction associations by TyG index using causal mediation analysis in subpopulations of 3 415 male participants ^a^

| Association component | Overweight (Ref. Under and normal weight) | | | |  | Obesity (Ref. Under and normal weight) | | | |
| --- | --- | --- | --- | --- | --- | --- | --- | --- | --- |
|  | HR (95% CI) | P value | Percentage of excess  association (95% CI) | P value |  | HR (95% CI) | P value | Percentage of excess  association (95% CI) | P value |
| Cardiovascular disease |  |  |  |  |  |  |  |  |  |
| Total association | 1.51 (1.25 to 1.82) | <0.001 | 100.0 |  |  | 2.21 (1.70 to 2.88) | <0.001 | 100.0 |  |
| Controlled direct association | 1.48 (1.21 to 1.80) | <0.001 | 93.8 (54.1 to 133.5) | <0.001 |  | 1.92 (1.40 to 2.64) | <0.001 | 75.9 (59.9 to 91.9) | <0.001 |
| Reference interaction ^b^ | -0.00 (-0.19 to 0.19) | 0.996 | -0.1 (-43.5 to 43.3) | 0.996 |  | 0.01 (-0.36 to 0.38) | 0.955 | 0.9 (-22.4 to 24.2) | 0.940 |
| Mediated interaction ^b^ | 0.00 (-0.12 to 0.12) | 0.993 | 0.1 (-23.6 to 23.8) | 0.993 |  | 0.23 (-0.10 to 0.55) | 0.176 | 18.6 (-8.0 to 45.2) | 0.171 |
| Pure indirect association | 1.03 (0.97 to 1.10) | 0.317 | 6.2 (-6.6 to 19.0) | 0.341 |  | 1.06 (0.95 to 1.18) | 0.317 | 4.6 (-4.9 to 14.1) | 0.342 |
| Stroke |  |  |  |  |  |  |  |  |  |
| Total association | 1.63 (1.21 to 2.19) | 0.001 | 100.0 |  |  | 2.40 (1.59 to 3.63) | <0.001 | 100.0 |  |
| Controlled direct association | 1.57 (1.14 to 2.17) | 0.006 | 88.1 (44.3 to 132.0) | <0.001 |  | 1.89 (1.12 to 3.18) | 0.016 | 61.5 (43.0 to 80.0) | <0.001 |
| Reference interaction ^b^ | -0.03 (-0.33 to 0.27) | 0.867 | -4.1 (-56.2 to 47.9) | 0.877 |  | 0.02 (-0.55 to 0.58) | 0.955 | 1.2 (-30.3 to 32.6) | 0.942 |
| Mediated interaction ^b^ | -0.07 (-0.26 to 0.13) | 0.509 | -10.5 (-43.7 to 22.8) | 0.538 |  | 0.22 (-0.28 to 0.71) | 0.390 | 15.4 (-17.6 to 48.4) | 0.360 |
| Pure indirect association | 1.17 (1.06 to 1.28) | 0.001 | 26.4 (0.1 to 52.8) | 0.049 |  | 1.31 (1.12 to 1.53) | 0.001 | 21.9 (0.8 to 43.0) | 0.042 |
| Heart disease |  |  |  |  |  |  |  |  |  |
| Total association | 1.42 (1.13 to 1.77) | 0.002 | 100.0 |  |  | 1.99 (1.45 to 2.74) | <0.001 | 100.0 |  |
| Controlled direct association | 1.42 (1.12 to 1.80) | 0.004 | 99.7 (32.8 to 166.6) | 0.003 |  | 1.90 (1.30 to 2.77) | 0.001 | 90.2 (63.2 to 117.3) | <0.001 |
| Reference interaction ^a^ | -0.01 (-0.22 to 0.21) | 0.954 | -1.5 (-71.6 to 68.6) | 0.967 |  | -0.01 (-0.45 to 0.44) | 0.977 | -0.7 (-35.9 to 34.6) | 0.971 |
| Mediated interaction ^a^ | 0.05 (-0.09 to 0.19) | 0.493 | 11.8 (-23.3 to 46.9) | 0.509 |  | 0.18 (-0.23 to 0.58) | 0.395 | 17.7 (-23.6 to 59.0) | 0.402 |
| Pure indirect association | 0.96 (0.89 to 1.03) | 0.276 | -10.0 (-29.3 to 9.3) | 0.311 |  | 0.93 (0.81 to 1.06) | 0.276 | -7.3 (-20.8 to 6.3) | 0.292 |

Abbreviations: HR, hazard ratio; CI, confidence interval; TyG, triglyceride-glucose.

^a^ Decomposition of total associations into controlled direct association (CDA), reference interaction (INTref), mediated interaction (INTmed), and pure indirect association (PIA) was done according to the 4-way decomposition causal mediation analysis method proposed by VanderWeele. CIs were calculated according to the delta method procedure. All models were adjusted for age, gender, marital status, residence, education level, smoking status, and drinking status as depicted in the directed acyclic graph (DAG).

^b^ INTref and INTmed are the estimation of additive excess relative risk due to interaction using HRs.

Table S4: Decomposition of the association of body mass index with incident cardiovascular disease including mediation and interaction associations by TyG index using causal mediation analysis in subpopulations of 6 884 participants with complete data ^a^

| Association component | Overweight (Ref. Under and normal weight) | | | |  | Obesity (Ref. Under and normal weight) | | | |
| --- | --- | --- | --- | --- | --- | --- | --- | --- | --- |
|  | HR (95% CI) | P value | Percentage of excess  association (95% CI) | P value |  | HR (95% CI) | P value | Percentage of excess  association (95% CI) | P value |
| Cardiovascular disease |  |  |  |  |  |  |  |  |  |
| Total association | 1.32 (1.17 to 1.49) | <0.001 | 100.0 |  |  | 1.93 (1.65 to 2.27) | <0.001 | 100.0 |  |
| Controlled direct association | 1.32 (1.16 to 1.50) | <0.001 | 100.1 (42.2 to 157.9) | 0.001 |  | 1.77 (1.47 to 2.14) | <0.001 | 83.0 (69.3 to 96.7) | <0.001 |
| Reference interaction ^b^ | 0.00 (-0.11 to 0.11) | 0.965 | 0.8 (-58.0 to 59.6) | 0.980 |  | -0.00 (-0.21 to 0.20) | 0.987 | -0.2 (-18.6 to 18.2) | 0.985 |
| Mediated interaction ^b^ | -0.06 (-0.12 to 0.01) | 0.093 | -17.3 (-39.0 to 4.5) | 0.120 |  | 0.07 (-0.11 to 0.25) | 0.453 | 7.3 (-11.6 to 26.3) | 0.448 |
| Pure indirect association | 1.05 (1.02 to 1.09) | 0.004 | 16.4 (2.4 to 30.4) | 0.022 |  | 1.09 (1.03 to 1.16) | 0.004 | 9.8 (2.3 to 17.4) | 0.011 |
| Stroke |  |  |  |  |  |  |  |  |  |
| Total association | 1.39 (1.13 to 1.73) | 0.002 | 100.0 |  |  | 1.99 (1.51 to 2.63) | <0.001 | 100.0 |  |
| Controlled direct association | 1.34 (1.07 to 1.69) | 0.011 | 86.0 (18.8 to 153.3) | 0.012 |  | 1.53 (1.08 to 2.16) | 0.017 | 52.2 (33.8 to 70.5) | <0.001 |
| Reference interaction ^b^ | -0.02 (-0.20 to 0.17) | 0.853 | -4.5 (-82.4 to 73.5) | 0.911 |  | 0.03 (-0.28 to 0.34) | 0.849 | 3.0 (-28.0 to 34.0) | 0.851 |
| Mediated interaction ^b^ | -0.05 (-0.16 to 0.06) | 0.332 | -13.8 (-44.7 to 17.1) | 0.382 |  | 0.22 (-0.05 to 0.49) | 0.115 | 22.1 (-3.1 to 47.3) | 0.086 |
| Pure indirect association | 1.13 (1.06 to 1.19) | <0.001 | 32.2 (3.0 to 61.4) | 0.031 |  | 1.23 (1.11 to 1.35) | <0.001 | 22.8 (5.5 to 40.1) | 0.010 |
| Heart disease |  |  |  |  |  |  |  |  |  |
| Total association | 1.31 (1.14 to 1.51) | <0.001 | 100.0 |  |  | 1.94 (1.62 to 2.32) | <0.001 | 100.0 |  |
| Controlled direct association | 1.32 (1.14 to 1.53) | <0.001 | 104.6 (32.9 to 176.4) | 0.004 |  | 1.94 (1.58 to 2.38) | <0.001 | 99.7 (83.3 to 116.1) | <0.001 |
| Reference interaction ^a^ | 0.01 (-0.12 to 0.13) | 0.922 | 2.0 (-68.4 to 72.5) | 0.955 |  | 0.00 (-0.26 to 0.26) | 0.988 | 0.2 (-20.8 to 21.2) | 0.984 |
| Mediated interaction ^a^ | -0.04 (-0.11 to 0.04) | 0.320 | -12.2 (-37.0 to 12.5) | 0.332 |  | -0.03 (-0.25 to 0.20) | 0.804 | -3.0 (-27.1 to 21.0) | 0.804 |
| Pure indirect association | 1.02 (0.98 to 1.06) | 0.410 | 5.6 (-8.2 to 19.3) | 0.427 |  | 1.03 (0.96 to 1.10) | 0.410 | 3.1 (-4.5 to 10.8) | 0.422 |

Abbreviations: HR, hazard ratio; CI, confidence interval; TyG, triglyceride-glucose.

^a^ Decomposition of total associations into controlled direct association (CDA), reference interaction (INTref), mediated interaction (INTmed), and pure indirect association (PIA) was done according to the 4-way decomposition causal mediation analysis method proposed by VanderWeele. CIs were calculated according to the delta method procedure. All models were adjusted for age, gender, marital status, residence, education level, smoking status, and drinking status as depicted in the directed acyclic graph (DAG).

^b^ INTref and INTmed are the estimation of additive excess relative risk due to interaction using HRs.

Table S5: Decomposition of the association of body mass index with incident cardiovascular disease including mediation and interaction associations by TyG index using causal mediation analysis in subpopulations of 6 723 participants with body mass index ≥18.5 kg/m^2^ ^a^

| Association component | Overweight (Ref. Normal weight) | | | |  | Obesity (Ref. Normal weight) | | | |
| --- | --- | --- | --- | --- | --- | --- | --- | --- | --- |
|  | HR (95% CI) | P value | Percentage of excess  association (95% CI) | P value |  | HR (95% CI) | P value | Percentage of excess  association (95% CI) | P value |
| Cardiovascular disease |  |  |  |  |  |  |  |  |  |
| Total association | 1.26 (1.12 to 1.43) | <0.001 | 100.0 |  |  | 1.89 (1.61 to 2.20) | <0.001 | 100.0 |  |
| Controlled direct association | 1.26 (1.11 to 1.43) | <0.001 | 100.0 (17.1 to 182.8) | 0.018 |  | 1.74 (1.45 to 2.09) | <0.001 | 83.7 (69.2 to 98.1) | <0.001 |
| Reference interaction ^b^ | -0.00 (-0.11 to 0.10) | 0.967 | -0.8 (-84.9 to 83.3) | 0.985 |  | 0.00 (-0.19 to 0.19) | 0.991 | 0.1 (-18.4 to 18.6) | 0.989 |
| Mediated interaction ^b^ | -0.05 (-0.11 to 0.01) | 0.119 | -17.8 (-42.5 to 6.8) | 0.157 |  | 0.06 (-0.10 to 0.22) | 0.473 | 6.7 (-11.4 to 24.7) | 0.469 |
| Pure indirect association | 1.05 (1.01 to 1.08) | 0.004 | 18.7 (1.6 to 35.8) | 0.033 |  | 1.08 (1.03 to 1.15) | 0.004 | 9.5 (2.1 to 17.0) | 0.012 |
| Stroke |  |  |  |  |  |  |  |  |  |
| Total association | 1.34 (1.08 to 1.66) | 0.007 | 100.0 |  |  | 2.02 (1.55 to 2.65) | <0.001 | 100.0 |  |
| Controlled direct association | 1.29 (1.03 to 1.62) | 0.027 | 83.7 (-1.6 to 168.9) | 0.054 |  | 1.64 (1.18 to 2.28) | 0.003 | 61.2 (43.2 to 79.3) | <0.001 |
| Reference interaction ^b^ | -0.02 (-0.19 to 0.16) | 0.846 | -5.1 (-104.0 to 93.7) | 0.919 |  | 0.03 (-0.29 to 0.34) | 0.874 | 2.5 (-25.0 to 29.9) | 0.861 |
| Mediated interaction ^b^ | -0.04 (-0.14 to 0.06) | 0.421 | -11.8 (-43.5 to 19.9) | 0.466 |  | 0.17 (-0.09 to 0.43) | 0.192 | 16.8 (-6.8 to 40.4) | 0.163 |
| Pure indirect association | 1.11 (1.06 to 1.18) | <0.001 | 33.2 (0.6 to 65.9) | 0.046 |  | 1.20 (1.10 to 1.31) | <0.001 | 19.5 (4.9 to 34.2) | 0.009 |
| Heart disease |  |  |  |  |  |  |  |  |  |
| Total association | 1.26 (1.09 to 1.45) | 0.001 | 100.0 |  |  | 1.85 (1.55 to 2.21) | <0.001 | 100.0 |  |
| Controlled direct association | 1.27 (1.10 to 1.47) | 0.001 | 104.1 (5.3 to 203.0) | 0.039 |  | 1.83 (1.49 to 2.24) | <0.001 | 96.8 (78.6 to 115.0) | <0.001 |
| Reference interaction ^a^ | 0.00 (-0.12 to 0.12) | 0.970 | 0.9 (-96.5 to 98.3) | 0.985 |  | -0.00 (-0.24 to 0.24) | 0.998 | -0.0 (-22.0 to 21.9) | 0.997 |
| Mediated interaction ^a^ | -0.03 (-0.10 to 0.04) | 0.389 | -11.6 (-38.9 to 15.6) | 0.403 |  | -0.00 (-0.20 to 0.20) | 0.987 | -0.2 (-23.3 to 22.9) | 0.987 |
| Pure indirect association | 1.02 (0.98 to 1.06) | 0.395 | 6.6 (-9.3 to 22.6) | 0.417 |  | 1.03 (0.96 to 1.10) | 0.394 | 3.4 (-4.6 to 11.4) | 0.407 |

Abbreviations: HR, hazard ratio; CI, confidence interval; TyG, triglyceride-glucose.

^a^ Decomposition of total associations into controlled direct association (CDA), reference interaction (INTref), mediated interaction (INTmed), and pure indirect association (PIA) was done according to the 4-way decomposition causal mediation analysis method proposed by VanderWeele. CIs were calculated according to the delta method procedure. All models were adjusted for age, gender, marital status, residence, education level, smoking status, and drinking status as depicted in the directed acyclic graph (DAG).

^b^ INTref and INTmed are the estimation of additive excess relative risk due to interaction using HRs.
